# Supplementary material for: Improved Real-Time Quaking Induced Conversion for Early Diagnostics of Creutzfeldt–Jakob Disease in Denmark
Source: Int J Mol Sci. 2023 Mar 23;24(7):6098. doi: 10.3390/ijms24076098 (PMC10094695; doi:10.3390/ijms24076098)
Supplement: Supplementary file 1 [file ijms-24-06098-s001.zip › Supplementary Figure 1 explainer.pdf]

Figure S1. Coomassie image of pooled fractions of rHaTrPrP, 90-231 aa (16.2 kDa). 1-3 is the collected, 4 is a thawed tube of rHaTrPrP after 1 week at 4 ^{o}C and 5 is the rHaFLPrP, 23-231 aa (22.9 kDa). Marker is depicted on the right.
