# Supplementary material for: Improved Real-Time Quaking Induced Conversion for Early Diagnostics of Creutzfeldt–Jakob Disease in Denmark
Source: Int J Mol Sci. 2023 Mar 23;24(7):6098. doi: 10.3390/ijms24076098 (PMC10094695; doi:10.3390/ijms24076098)
Supplement: Supplementary file 1 [file ijms-24-06098-s001.zip › Supplementary figure 2 explainer.pdf]

Figure S2. Mean of two best replicates (individual black lines) for all CSF samples (*n*=18) with probable CJD. Red is a mean of all negative replicates in a quadruple set-up. Dotted line represents the RFU cut-off value for a positive replicate.
