# Supplementary figures and images for: Improved Real-Time Quaking Induced Conversion for Early Diagnostics of Creutzfeldt–Jakob Disease in Denmark
Source: Int J Mol Sci. 2023 Mar 23;24(7):6098. doi: 10.3390/ijms24076098 (PMC10094695; doi:10.3390/ijms24076098)

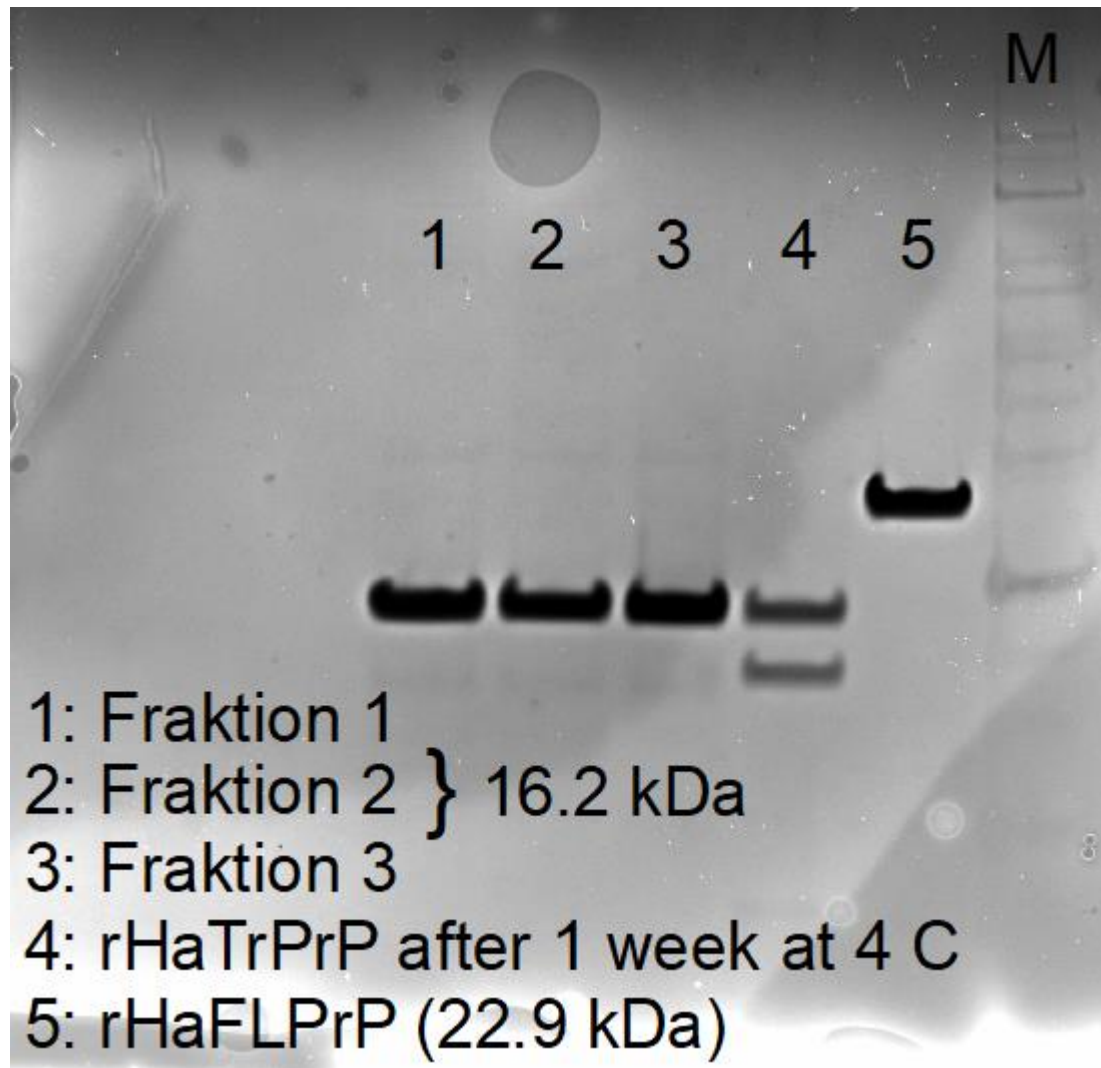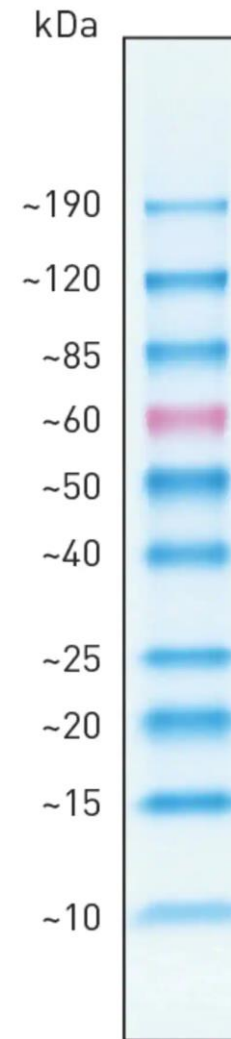

Supplement: Supplementary file 1 [file ijms-24-06098-s001.zip › Supplementary Figure 1.pdf]

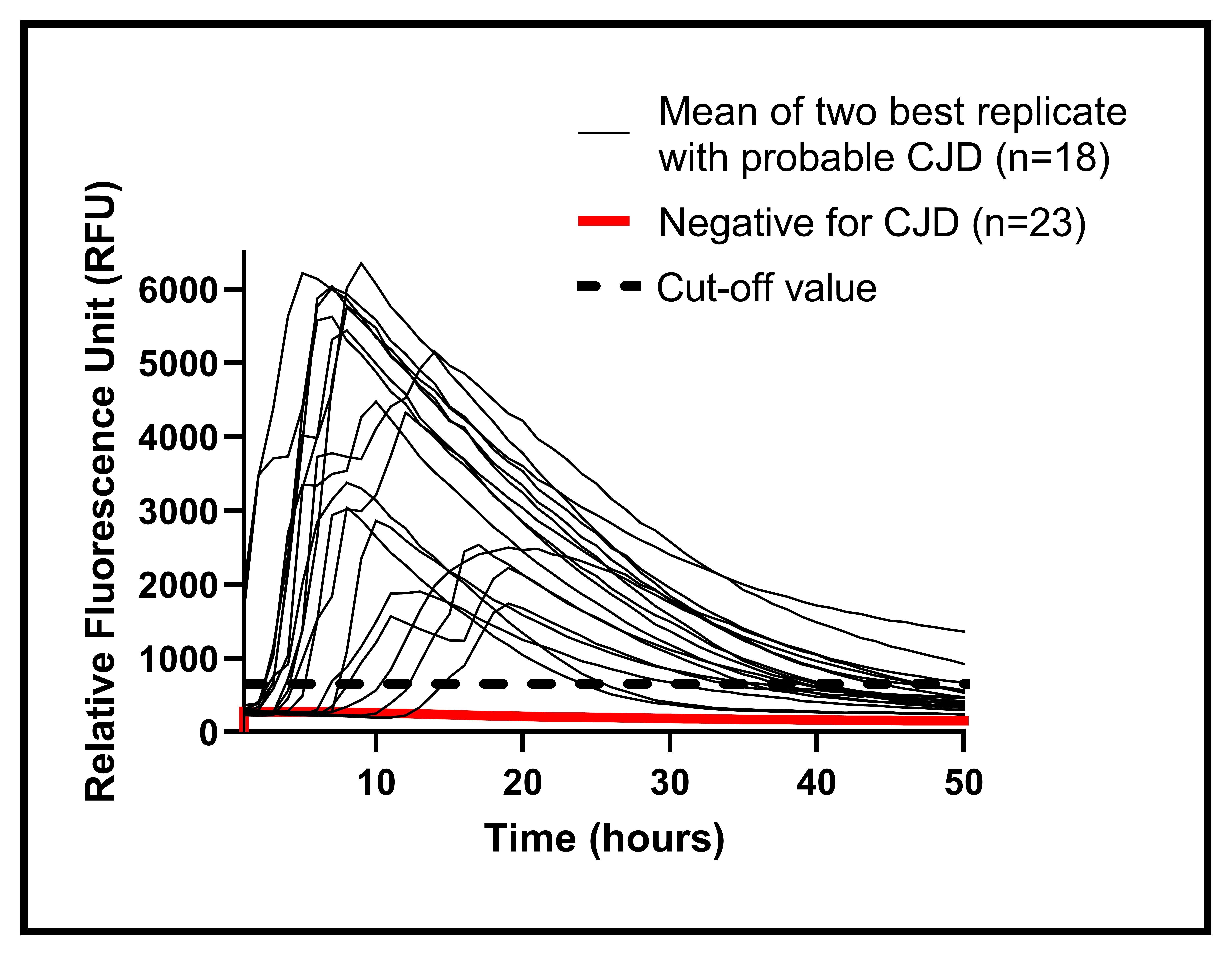

Supplement: Supplementary file 1 [file ijms-24-06098-s001.zip › Supplementary Figure 2.jpg]
